# Innate Immune Activity in Glomerular Podocytes

**DOI:** 10.3389/fimmu.2017.00122

**Published:** 2017-02-08

**Authors:** Hong Xia, Wenduona Bao, Shaolin Shi

**Affiliations:** ^1^National Clinical Research Center for Kidney Diseases, Jinling Hospital, Nanjing University School of Medicine, Nanjing, China; ^2^Department of Nephrology, Zhejiang Provincial Hospital of Traditional Chinese Medicine, Hangzhou, China

**Keywords:** innate immunity, mitochondrial DAMPs, podocyte injury, apoptosis, lupus nephropathy

## Abstract

Glomerular podocytes are specialized in structure and play an essential role in glomerular filtration. In addition, podocyte stress can initiate glomerular damage by inducing the injury of other glomerular cell types. Studies have shown that podocytes possess the property of immune cells and may be involved in adaptive immunity. Emerging studies have also shown that podocytes possess signaling pathways of innate immune responses and that innate immune responses often result in podocyte injury. More recently, mitochondrial-derived damage-associated molecular patterns (mtDAMPs) have been shown to play a critical role in a variety of pathological processes in cells. In the present mini-review, we summarize the recent advances in the studies of innate immunity and its pathogenic role in podocytes, particularly, from the perspective of mtDAMPs.

Podocytes are highly specialized in structure and characterized by abundant foot processes between which slit diaphragms are formed and serve as part of glomerular filtration barrier. Podocyte homeostasis is critical for optimal glomerular filtration and podocyte injury or loss can initiate glomerular damage, possibly leading to glomerulosclerosis.

Podocytes exhibit features of immune cells. For example, podocytes express certain genes characteristic of immune cells, including MHC class II (1), B7-1 (CD80, a molecule required for T cell activation) (2), and FcRn (a receptor protein in antigen-presenting lymphocytes) (3). It has been demonstrated that podocytes can act as antigen-presenting cells as shown by their capabilities of phagocytosing and processing antigens and presenting them by forming peptide–MHC complexes, which can activate T cells (4). Importantly, this property of podocytes as antigen-presenting cells is involved in the development of nephritis as shown by amelioration of nephritis in the mice lacking MHC II specifically in podocytes in an anti-GBM nephritis model (4). Recently, increasing numbers of studies have shown that podocytes possess the components of innate immunity. The innate immune responses induced by various ligands have pathological role, leading to podocyte injury and podocytopathies. We will review the studies in this area and will discuss mitochondrial damage-associated molecular patterns (DAMPs) and their involvement in the innate immune activity in podocytes.

## Innate Immune Responses in Podocytes

### Innate Immune System in Podocytes

Podocytes express many genes required for innate immune responses. These genes include a number of pattern recognition receptors (PRRs) that sense both pathogen-associated molecular patterns (PAMPs, e.g., microbial pathogens) and DAMPs (e.g., endogenous molecules in an organism). In addition, the components of downstream signaling pathways are also present in podocytes.

#### Toll-Like Receptors (TLRs)

Most members of the TLR family are detectable at mRNA level in isolated mouse glomeruli with TLR3 and TLR4 having the most abundant expression (5). Immunohistochemical staining showed that TLR4 protein is localized in podocytes. In cultured podocytes, the treatment with lipopolysaccharides (LPS), a PAMP ligand for TLR4, induced the expression of CCL2, CCL7, CXCL1, CXCL5, CCL3, CCL5, CXCL7, CXCL9, CXCL11, and CXCL13. Moreover, fibrinogen, a DAMP ligand of TLR4, also induced the expression of CCL2, CCL7, CXCL1, and CXCL5 (5). Consistently, nuclear factor kappa B (NFκB) signaling was found activated in podocytes that were treated with TLR ligands (6). Supportively, the essential components of TLR signaling in cultured podocytes, including MyD88, IRAK, TRAF6, etc., have also been detected (7). In patients with lupus nephritis, polyoma virus infection, or hemolytic uremic syndrome, TLR9 was found to be *de novo* expressed in podocytes and thought to be involved in immune response and inflammation in glomeruli (8–10).

#### NLRP3 and Inflammasomes

The major components of inflammasomes, NLRP3, ASC, and caspase 1, were found to be expressed in podocytes (11), and PAMP treatment was capable of upregulating the expression of NLRP3, pro-caspase 1, posttranslational processing of pro-caspase 1, and the release of IL-18 in podocytes (12). Cellular endogenous reactive oxygen species (ROS) was observed to induce the activation of NLRP3 and formation of inflammasomes in mouse podocytes and glomeruli (13).

#### Retinoic Acid-Inducible Gene 1 (RIG-I)

Retinoic acid-inducible gene 1 belongs to the family of RIG-I-like helicases, which recognize viral RNA. It is expressed in podocytes and fully functional *via* downstream pathways, IRF3 (transcription factor interferon regulatory factor 3), and NFκB, leading to cytokines expression and podocyte injury (14).

#### Receptor of Advanced Glycation Endproducts (RAGE)

Receptor of advanced glycation endproducts is one of the PRRs and can use DAMPS as its ligands. These DAMPs include advanced glycation endproducts (AGE), high-mobility group box protein 1 (HMGB1), S100A proteins, etc. RAGE is expressed in podocytes and upregulated in diabetic nephropathy (DN) (15, 16), and mediate podocyte injury induced by AGE (17). More recently, advanced oxidation protein products were also shown to be ligands of RAGE and capable of inducing podocyte apoptosis (18).

### Innate Immune Responses Are Associated with Podocyte Injury

Although innate immune signaling pathways are present and can be activated in podocytes, there has been no study showing that the innate immune responses enable podocytes to eliminate pathogens in the body. Instead, a number of reports have described that innate immune responses lead to podocyte injury.

It was shown that injection of a TLR4 ligand, LPS, into mice induced proteinuria, accompanied by podocyte foot process effacement, in 24 h (2). This effect of LPS requires the expression of B7-1 (CD80) in podocytes because LPS does not induce proteinuria in B7-1 knockout mice (2). TLR3 and RIG-I were shown to mediate the effects of viruses on podocytes, leading to structural and functional disturbance of podocytes (14). This conclusion was further supported by the observation that the treatment with polyIC, a TLR3 ligand, caused proteinuria and changes in podocytes (19). TLR signaling is also involved in podocyte injury in DN by mediating the effect of serum LPS on podocytes (20).

Apoptosis is a major consequence of podocyte injury. Intrinsic caspase 3 pathway was shown to be responsible for podocyte apoptosis. However, it has just been shown that it is caspase 1, but not caspase 3, that mediates podocyte injury, at least, in DN, and that this process involves NLRP3/inflammasomal pathway (21). Involvement of NLRP3/inflammasomes was also demonstrated in other podocyte injury model (22). This finding is unexpected and surprising and has led to paradigm shift regarding the mechanism underlying podocyte death. It is important to further investigate how NLRP3/inflammasomes are activated and what the responsible ligands are.

## Mitochondrial DAMPs (mtDAMPs)

Mitochondria are believed to have evolved from ancient bacteria through endosymbiogenesis. They, therefore, have their own DNA and protein synthesis machinery. Mitochondrial DNA (mtDNA) and the proteins synthesized in mitochondria are both different from nuclear DNA (nDNA) and the proteins synthesized on ER.

### Formyl Peptides

Like bacteria, mitochondria use formylmethionine (methionine attached with a formyl group) for the initiation of protein synthesis, leaving the synthesized proteins formyl modified. During evolution, eukaryotic organisms developed formyl-peptide receptors that recognize bacterial formyl peptides, leading to innate immune responses and bacterial clearance. It is known that mitochondrial formyl peptides are still capable of inducing innate immune responses (23).

### Mitochondrial DNA

Mitochondrial DNA retains the features of bacterial DNA, including CpG unmethylation. Like bacterial DNA, which acts on TLR9 to induce innate inflammatory response or NLRP3 to produce proinflammatory cytokine in immune cells (24, 25), mtDNA has been shown to act on TLR9 and NLRP3 similarly in macrophages (26–28).

### Mitochondrial Transcription Factor A (Tfam)

Mitochondrial transcription factor A is a nuclear gene-encoded protein that is functionally and structurally homologous to HMGB1, a known endogenous danger molecule, and has been shown to act as a danger molecule as well. It can work with unmethylated CpG DNA to act on RAGE and induce response of plasmacytoid dendritic cells, leading to release of TNF-α (29, 30). In addition, recombinant Tfam treatment can induce TNF-α expression in cultured macrophages, as well as inflammatory responses in animals (31).

## mtDAMPs Induce Innate Immune Responses and Injury in Podocytes

Podocytes possess many PRRs that are functional as discussed above. Particularly, podocytes express TLR9 and NLRP3 that can use mtDNA as ligand, as well as RAGE that can use Tfam as ligand. These suggest that mtDAMPs may act on podocytes to activate innate immune pathways.

### Endogenous mtDNA and TLR9 Mediate Puromycin Aminonucleoside (PAN)-Induced Podocyte Injury

Puromycin aminonucleoside has recently been shown to be capable of inducing mtDNA translocation to lysosomes in which mtDNA acts as ligand to stimulate TLR9 in podocytes, thereby enhancing proapoptotic NFκB and p38 MAPK signaling, inflammatory cytokine expression, and apoptosis level (7, 32). Nevertheless, mtDNA may also act through NLPR3/inflammasomes/caspase 1 pathway in the experimental system because of the presence of the NLRP3–caspase 1 pathway in podocytes (11). As already mentioned earlier, caspase 1 may be more important than caspase 3 in podocyte apoptosis, at least, in the condition of diabetes (21).

### mtDNA–NLRP3 Interaction May Underlie Podocyte Injury in DN

We speculate that mtDNA–NLRP3 interaction may be an important mechanism underlying podocyte injury/apoptosis in DN. It is known that high glucose can induce ROS in podocytes, as well as apoptosis of the cells (33), and ROS can induce mtDNA oxidative damage in podocytes (34). Importantly, oxidized mtDNA has been shown to be more potent in activating NLRP3, inflammasomes, and apoptosis in macrophages (27). Together with the observation that caspase 1, but not caspase 3, mediates podocyte injury in DN (21), these studies strongly suggest that high glucose or hyperglycemia may cause podocyte injury or death through an innate immune pathway involving NLRP3 in diabetes.

### AGE–RAGE Interaction May Also Contribute to Podocyte Injury in DN

Innate immune responses can also be induced by AGE through interaction with RAGE and lead to podocyte injury. Under diabetic condition, it was found that a high level of AGE (e.g., glycated albumin) can cause podocyte apoptosis *via* RAGE, leading to innate immune response and podocyte apoptosis (35).

### mtDAMPs May Play an Important Role in Lupus Nephropathy (LN)

Systemic lupus erythematosus (SLE) is a type of autoimmune disease in which the immune system of the patient becomes hyperactive and attacks normal tissues, including kidney. In the development of SLE and LN, mtDAMPs likely play a role described as follow.

#### mtDNA/TLR9 May Be Involved in the Development of SLE and LN

In SLE-prone MRL-Fas(lpr) mice, the treatment with DNA derived from bacteria or CpG ODN facilitated the development of SLE and LN in the mice through activating TLR9 in B cells (36, 37). The treated mice manifested an increase of DNA autoantibodies, cytokines, or chemokines in circulation, and infiltration of immune cells in tissues, including kidney.

In addition to B cells, mtDNA–TLR9 may also facilitate SLE development through plasmacytoid predendritic cells (PDCs) as there has been a study showing that viral DNA or the DNA in the immune complexes can stimulate PDCs through TLR9 to express and secrete IFN-α, a cytokine that is involved in anti-dsDNA antibody production (38). Meanwhile, mtDNA has been shown to have antigenicity that contributes to the formation of anti-dsDNA antibodies. Interestingly, oxidized form of mtDNA exhibited an enhanced antigenicity compared to that not oxidized (39). Additionally, the autoantibodies in SLE patients are reactive to mtDNA and have a higher affinity with mtDNA than nDNA (40, 41). Consistently, the level of anti-mitochondrial antibody is correlated with the development of SLE (42). These observations suggest that mtDNA and TLR9 are involved in the development of SLE and LN.

#### mtDNA/TLR9 May Contribute to Podocyte Injury in LN

Puromycin aminonucleoside is capable of inducing IFN-α expression in podocytes *via* mtDNA and TLR9 interaction (7). In addition, several studies have shown that TLR9 is *de novo* expressed in podocytes in a proportion of SLE patients (8–10). This raises a possibility that podocytes are an alternative source of IFN-α and other cytokines that are involved in SLE and LN development. SLE is characterized by the production of anti-double stranded (ds) DNA antibodies, which bind to dsDNA to form immune complexes (ICs). Such DNA-containing ICs are capable of stimulating plasmacytoid PDCs to express IFN-α through TLRs, including TLR9 (38), it is thus reasonable to speculate that the anti-dsDNA antibodies can also bind mtDNA to form ICs. Since lupus nephritis is pathologically characterized by the deposition of the ICs in glomeruli, we speculate that the mtDNA in the ICs may be transported to podocytes through endocytosis and then either reach endolysosomes to activate the *de novo* expressed TLR9 or act on NLRP3/inflammasomes, resulting in podocyte injury/apoptosis and IFN-α and proinflammatory IL-1 production thereby facilitating LN development. To prove this hypothesis, it would be important to examine whether the ICs in lupus contain mtDNA.

As having mentioned above that TLR9 *de novo* expression in podocytes occurs in some of the patients of LN with urinary protein level of 0.3–0.5 g/24 h or a negative urinary protein (9), it would be interesting to investigate the consequences of TLR9 *de novo* expression in podocytes in the condition. Hu and colleague have recently reported that podocytopathy in the LN patients with nephrotic range of proteinuria can be divided into two types, MCD/mesangial proliferation and FSGS, which differ in pathology, tubular lesion, and treatment outcome (43). Whether TLR9 expression underlies the difference of the two types of lupus podocytopathy warrants further investigation. One feasible approach for the study could be to perform immunohistochemical staining of TLR9 with renal biopsies in a cohort of LN consisting of patients with MCD/mesangial proliferation and FSGS; and then test whether TLR9 *de novo* expression in podocytes correlates with the patients with FSGS, which manifests with podocyte injury and loss in contrast with MCD.

## Circulating mtDAMPs and Podocyte Inflammation and Injury

Proteinuria is frequently observed in the patients with trauma and burning and the resulting sterile systemic inflammation response syndrome (SIRs) (44–47). Interestingly, elevation of circulating mtDNA level has also been observed in the patients (48–52). It is thus possible that mtDNA in circulation may play a pathologic role in proteinuria development through damaging podocytes. Moreover, since the increase of mtDNA in circulation should be accompanied by other mtDAMPs, e.g., Tfam, it is also possible that these mtDAMPs may act in concert to damage podocytes. One study has shown that injection of mtDAMPs into rats *via* tail can cause transient mild proteinuria several hours following the injection (32). This little effect of mtDAMPs on podocytes may be due to the fact that TLR9 is not expressed in podocytes of normal rats, thus precluding the action of mtDNA. Thus, the mtDAMPs other than mtDNA might be responsible for the mild proteinuria in the rats. Whether a long-term high level of mtDAMPs in circulation would cause more severe podocyte injury, proteinuria and even glomerulosclerosis would be interesting to explore.

## Concluding Remarks

The available studies have shown that podocytes possess many features of immune cells of both adaptive and innate immunity. As non-immune cells, podocytes are usually injured by the innate immune responses induced by PAMPs or DAMPs (including mtDAMPs). Therefore, further studies of innate immune responses in podocytes would provide more insights into the mechanism underlying podocyte injury, likely leading to improved therapeutics and diagnostics for podocytopathies.

## Author Contributions

HX: collected literature and wrote manuscript; WB: collected literature and discussed the manuscript; SS: chose the topic, collected literature, and wrote manuscript.

## Conflict of Interest Statement

The authors declare that the research was conducted in the absence of any commercial or financial relationships that could be construed as a potential conflict of interest.
